# miRAS: a data processing system for miRNA expression profiling study

**DOI:** 10.1186/1471-2105-8-285

**Published:** 2007-08-04

**Authors:** Feng Tian, Huayue Zhang, Xinyu Zhang, Chi Song, Yongjing Xia, Yiqing Wu, Xiangjun Liu

**Affiliations:** 1 Department of Biological Science and Biotechnology, Tsinghua University, Beijing 100084, People's Republic of China; 2 Institutes of Biomedicine, Tsinghua University, Beijing 100084, People's Republic of China; 3 Ministry of Education Key Laboratory of Bioinformatics, Tsinghua University, Beijing 100084, People's Republic of China

## Abstract

**Background:**

The study of microRNAs (miRNAs) is attracting great considerations. Recent studies revealed that miRNAs play as important regulators of gene expression and some even as cancer players or inhibitors. Many studies try to discover new miRNAs and reveal the miRNA expression profile in cancer using a SAGE-based total RNA clone method. However, the data processing of this method is labor-intensive with several different biological databases and more than ten data processing steps involved.

**Results:**

With miRAS, miRNAs and possible miRNA candidates contained in the submitted sequencing data were obtained together with their expression profile. The functions of known and predicted miRNAs were then analyzed by miRNA target prediction followed by target gene annotations. Finally, to extract the biological significance of the miRNAs in the samples, further annotations of the known miRNA and target genes were performed by collecting the public expression datasets of miRNA and target genes in normal and cancer tissues.

**Conclusion:**

We introduce a web-based analysis platform called miRNA Analysis System (miRAS), for processing and analyzing of the sequence data obtained from the total RNA clone method. The system was built on generalizing the study of a liver cancer cell line total RNA sequencing project. miRAS is freely available on the web.

## Background

MicroRNAs (miRNAs) are a class of small non-coding RNAs that regulate gene expression by binding to their target mRNAs and triggering either protein translation repression or RNA degradation [1]. Recent studies show that some miRNAs are located at fragile sites and genomic regions involved in cancers [2]. The aberrant expression of miRNA genes could lead to human disease, including cancer [3-6], and are regarded as potential biomarkers for cancer diagnosis [7,8]. The roles miRNAs play have been demonstrated in a few cancer types including breast cancer [9], lung cancer [10] and chronic lymphocytic leukemia [2,11,12], while the roles of miRNA in other cancers remain largely unknown.

There are several approaches of studying miRNAs and their expression profiles, including Northern blotting and real-time PCR assay. There are also available high-throughput methods such as oligonucleotide miRNA microarray analysis [13-15], bead-based flow-cytometric technique [8], and SAGE-based miRAGE [16]. miRNA microarray analysis is a commonly used high-throughput technique for the assessment of previously discovered miRNAs. With the SAGE-based technique, such as miRAGE, the expression profiles of known miRNAs could be retrieved together with the unknown ones which are possible miRNA candidates.

For gene expression SAGE studies [17,18], there exist several well developed methods for data analysis together with web services provided, such as SAGEmap [19] and SAGE Genie [20]. For miRNA-related SAGE, however, the data analysis is much more complicated. The extracted tags have to be compared with various RNA databases in addition to mRNA sequences. The tags also need to be mapped to the human genome and to be analyzed for precursors with thermodynamically stable hairpin structures. This is a very troublesome process and current users have to refer to several different databases to retrieve related biologically significant data [16]. To aid the processing and data analysis of this method, we constructed a web-based system, named miRNA Analysis System (miRAS). The expression profile of known miRNAs in submitted sequences were returned and compared with public dataset using Fisher's exact test. Public available datasets of known miRNAs expression in liver were collected for the annotation of miRNA expression in liver. Several public available gene expression datasets were included to reveal differentially expressed genes in liver cancer and normal liver tissues. The differentially expressed miRNAs and genes are highlighted and the relationship between miRNAs and genes is shown according to miRNA target prediction.

## Results and Discussion

Users could upload the raw sequencing data and specify the sequencing parameters through the web interface. The known miRNAs and possible miRNA candidates will be analyzed together with their expression profiles. The target genes predicted by miRNA target prediction software are provided together with the annotation information. To demonstrate the biological significance of the retrieved miRNAs, the profiles of public datasets of known miRNAs and target genes were collected and included in the annotation.

The miRAS system provides an easy and friendly way for scientists to analyze and process raw miRNA sequence data to obtain new miRNA candidates. It also provides tools for the annotations of the predicted miRNAs.

## Conclusion

In this work, we established a web-based analysis platform for miRNAs, called miRAS [21], to analyze the miRNA expression in specific tissue and to predict and study the possible miRNA candidates. The differentially expressed miRNAs that target differentially expressed genes are retrieved together with miRNA and target gene annotation, to uncover the biological significance. Currently it supports liver cancer genes, while in the future, the analysis platform is planned to be expanded to support other cancers and to integrate all public available expression data of the miRNAs and genes in cancer and normal tissues.

## Methods

**Figure 1:**
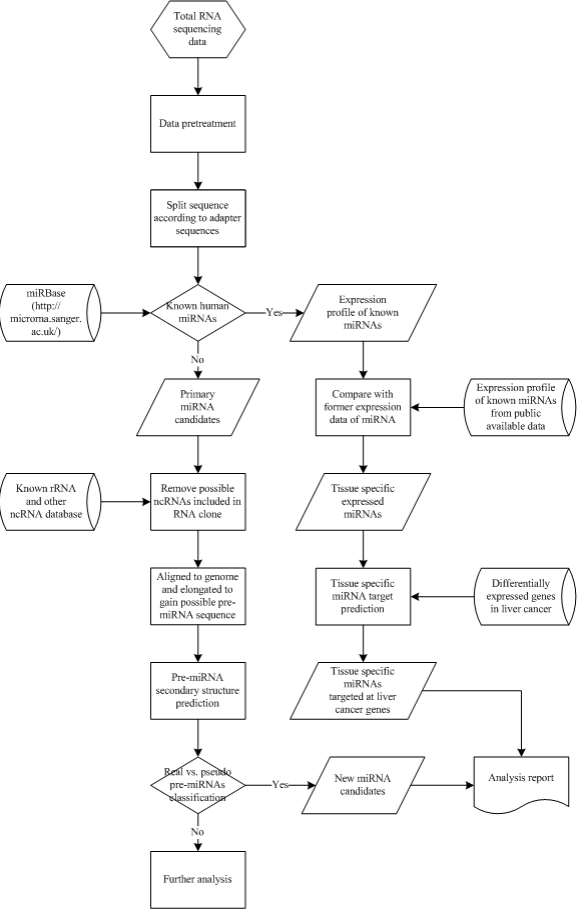
Work flow of miRAS.

The work flow of miRAS is diagrammed in Figure 1. Details of the major steps  of the system are described in the following sections.

### Preliminary processing of sequence data

Multiple sequence data of RNA sequencing in multi-fasta format could be uploaded for analysis directly. The raw sequencing data in trace file format could be compressed in tar, tar.gz, zip, or rar format, and uploaded.

If the trace data of total RNA sequencing result is selected, the sequence and quality of the sequence data will be checked by PHRED [22,23], which reads DNA sequence trace data, calls bases, assigns quality values to the bases, and writes the base calls and quality values to output files. Sequences with low quality base call values, to be removed, are detected by PHRED with default parameters, which explore modified Mott trimming algorithm, an error probability cutoff value of 0.05 and a minimum segment length of 20 bases. The produced high-quality reads are then compared to the vector sequence consensus using the cross-match program, the mostly used software of finding vector regions on sequencing reads. Parameters of -minmatch 12 -minscore 20 are used as default, while users could also apply their own parameters through the web interface. A standard vector database provided by PHRAP package is used as default and it can also be specified by the users. The detected vector sequences contained in the sequence reads will be removed. The adapter sequences contained in the miRNA clone sequences are checked and removed similarly, using the cross-match program with parameters (-minmatch 8 -minscore 14) for combined adaptors and (-minmatch 8 -minscore 8) for single adapters.

### Extraction of known miRNAs and primary new candidate miRNAs

After removal of the adapter sequences, redundant RNA segments are removed and the copy numbers of each segment are recorded. The resulting short RNA segments are searched against a known human miRNA dataset (miRNA Registry Release 9.1) [24,25] to retrieve known miRNAs. The expressions of the known miRNAs in given tissues are compared with public expression data to obtain differentially expressed or tissue specifically expressed miRNAs. Other non-coding RNA (ncRNA) are identified in a similar way, by searching against a ncRNA database, which was built by combining several public RNA databases, including rRNA database from Ribosomal Database Project (RDP) [26], ncRNA database from NONCODE [27] and Regulatory noncoding RNAs database [28]. 1 nt miss match is allowed in retrieving the blast output for the tolerance of mistake in DNA sequencing. Those not included in the known miRNAs or other ncRNA databases are treated as primary miRNA candidates.

### Pre-miRNA retrieval and secondary miRNA candidates screening

The primary miRNA candidates identified from the previous step are aligned to the human genome. For each aligned segment, the 99 nucleotides upstream to the alignment position on the genome, the sequence of the primary candidate miRNA itself, and the 99 nucleotides downstream to it on the genome are concatenated and used as a possible precursor sequence of the primary candidate. The secondary structures of the precursor sequences are predicted with the mfold program [29] and RNAfold [30], and checked with two criteria of miRNA precursors: 1) whether the candidate precursor sequence has a characteristic hairpin secondary structure and 2) whether a single miRNA molecule accumulates one arm of a hairpin precursor molecule [31]. Support vector machine (SVM) [32] is applied to classify real vs. pseudo pre-miRNAs.

### miRNA target prediction and function annotation

The 3' untranslated region (3' UTR) of known human genes were retrieved from UCSC website. The most commonly used miRNA target scan program for mammalian, miRanda program [33], is used to predict the target genes of the known miRNAs and miRNA candidates. To demonstrate the biological significance of the target genes, several datasets of microarray were studied to obtain differentially expressed genes in normal and cancer tissues. The results are shown together with the target gene annotations.

The expression profiles of the miRNAs are included in the analyses to reveal the regulatory roles of the miRNAs. The expression data of normal liver tissue miRNAs were obtained from the RNA Project at Rockefeller [34]. The expression of known miRNAs retrieved from users' clone data are compared with that in normal liver using Fisher's exact test to find differentially expressed miRNAs in users' RNA clone and normal liver tissues.

To further view expression profiles of known miRNAs in liver, public available expression datasets of miRNA in liver were collected and processed. Different types of miRNA expression data, such as SAGE, microarray and bead-base array, were processed with different methods. For SAGE based data, Tags per million (TPM) of a miRNA, regarded as the expression level of this miRNA, is calculated and shown in our web display. For bead-base array and microarray, on the other hand, the log ratio value (M value) is normally directly provided by the data source. In some cases, several M values were provided and the average values were computed in our study. The M value represents overexpression or underexpression level of each miRNA, and is shown in the web interface.

The expression data of the target genes of known miRNAs were also provided. The microarray datasets were processed with the siggenes module of the R package from Bioconductor [35] which implements the algorithm of Significance Analysis of Microarrays (SAM) proposed by Tusher et al [36], using a modified t statistics by scoring each gene on the basis of change in gene expression relative to the standard deviation of repeated measurements. For SAGE datasets, SAGE tags were mapped to known genes with the data provided by NCBI ftp site [37]. The differential expression between cancerous cell and normal cell was analyzed using Fisher's exact test [38-40], implemented with the sagenhaft module of R. For both types, M values were computed to represent the differential expression of the genes for cancerous vs. normal tissues.

The expression datasets of known miRNAs and their target genes included in miRAS are listed in Table 1 and Table 2.

**Table 1:** Liver miRNA expression datasets in miRAS

Provider	Dataset Type	Tissue Description
Rockefeller Univ.*	SAGE	Mouse liver tissue
Shingara, J., et al. **	Microarray	Mouse liver tissue
Farh, K.K., et al.**	Microarray	Mouse liver tissue
Thomson, J.M., et al.***	Microarray	Mouse liver tissue
Lu, J., et al.***	Bead-base array	Human normal liver
Wienholds, E., et al.***	Microarray	Zebrafish tissues

* Datasets from the Rockefeller University RNA project [34].

** Datasets available from supplements of article, references [41] and [42], respectively.

*** Datasets available from Gene Expression Omnibus (GEO) [43] with accession number of GSE1635, GSE2564, and GSE2628, respectively.

**Table 2:** Liver gene expression datasets in miRAS

Dataset Type	Dataset Name	Tissue Description
Microarray:		
	Hep3B vs Universal_control*	Hepatoma cell line
	SNU_387 vs Universal_control*	Hepatoma cell line
	HepG2 vs Universal_control*	Hepatoma cell line
	PLC_PRF_5 vs Universal_control*	Hepatoma cell line
	SNU_182 vs Universal_control*	Hepatoma cell line
	SNU_354 vs Universal_control*	Hepatoma cell line
	SNU_368 vs Universal_control*	Hepatoma cell line
	SNU_449 vs Universal_control*	Hepatoma cell line
	SNU_475 vs Universal_control*	Hepatoma cell line
SAGE:		
	SAGE_Liver_cholangiocarcinoma_B_K1**	Liver
	SAGE_Liver_cholangiocarcinoma_B_K2D**	Liver
	SAGE_Liver_cholangiocarcinoma_CL_K3**	Liver cell line
	SAGE_Liver_cholangiocarcinoma_CL_K4**	Liver cell line
	SAGE_Liver_normal_B_1**	Normal bulk liver

* Datasets from GEO [43], GEO accession GSM81024- GSM81059

** Datasets from SAGE Genie [44] of CGAP, names are for library.

### Result retrieval

Through the result view page, the base-calling result, known mRNAs and other ncRNAs, possible miRNA candidates together with the secondary structures, target prediction result and their expressions in known datasets are returned. For users to view result in more convenience, different data formats are provided, such as raw data file, text file and Microsoft excel.

## Competing interests

The authors declare that they have no competing interests.

## Availability

miRAS is freely available on the web [21] suitable for most graphical web browser. User registered with an email address will be alerted the status of the submitted jobs. The system is also anonymously accessible.

## Authors' contributions

FT proposed the analysis pipeline and completed the main part of this research. HZ implemented the CGI scripts and the system testing. SC and XZ helped in the webpage writing and the database for gene annotation. XZ also created a database for the processing of the SAGE data. YX, YW helped to review the manuscripts. XL supervised the design and implementation of the system, and advised on the manuscript preparation.
